# Association analysis of a highly polymorphic CAG Repeat in the human potassium channel gene *KCNN3 *and migraine susceptibility

**DOI:** 10.1186/1471-2350-6-32

**Published:** 2005-09-14

**Authors:** Robert Curtain, James Sundholm, Rod Lea, Mick Ovcaric, John MacMillan, Lyn Griffiths

**Affiliations:** 1 Genomics Research Centre, School of Health Science, Griffith University, Gold Coast, Queensland, Australia; 2 Institute of Environmental Science and Research, Wellington, New Zealand; 3 Queensland Clinical Genetics Service, Royal Children's Hospital Health Service District, Brisbane, Queensland, Australia

## Abstract

**Background:**

Migraine is a polygenic multifactorial disease, possessing environmental and genetic causative factors with multiple involved genes. Mutations in various ion channel genes are responsible for a number of neurological disorders. *KCNN3 *is a neuronal small conductance calcium-activated potassium channel gene that contains two polyglutamine tracts, encoded by polymorphic CAG repeats in the gene. This gene plays a critical role in determining the firing pattern of neurons and acts to regulate intracellular calcium channels.

**Methods:**

The present association study tested whether length variations in the second (more 3') polymorphic CAG repeat in exon 1 of the *KCNN3 *gene, are involved in susceptibility to migraine with and without aura (MA and MO). In total 423 DNA samples from unrelated individuals, of which 202 consisted of migraine patients and 221 non-migraine controls, were genotyped and analysed using a fluorescence labelled primer set on an ABI310 Genetic Analyzer. Allele frequencies were calculated from observed genotype counts for the *KCNN3 *polymorphism. Analysis was performed using standard contingency table analysis, incorporating the chi-squared test of independence and CLUMP analysis.

**Results:**

Overall, there was no convincing evidence that *KCNN3 *CAG lengths differ between Caucasian migraineurs and controls, with no significant difference in the allelic length distribution of CAG repeats between the population groups (*P *= 0.090). Also the MA and MO subtypes did not differ significantly between control allelic distributions (*P *> 0.05). The prevalence of the long CAG repeat (>19 repeats) did not reach statistical significance in migraineurs (*P *= 0.15), nor was there a significant difference between the MA and MO subgroups observed compared to controls (*P *= 0.46 and *P *= 0.09, respectively), or between MA vs MO (*P *= 0.40).

**Conclusion:**

This association study provides no evidence that length variations of the second polyglutamine array in the N-terminus of the *KCNN3 *channel exert an effect in the pathogenesis of migraine.

## Background

Migraine is a common, debilitating neurovascular disease characterised by severe recurrent headache, nausea and vomiting, photophobia and phonophobia [1]. It is clinically diagnosed based on criteria specified by the International Headache Society (IHS), defining two major classes of migraine: migraine with aura (MA) and migraine without aura (MO). MA sufferers experience neurovascular disturbances that precede the headache phase of an attack. Although migraine is partly influenced by environmental determinants, there is a significant genetic component, with disease heritability estimated to be up to 60% [2] and mode of transmission multifactorial. The disorder is common with a large Dutch study reporting lifetime prevalence estimates of 33% in women and 13.3% in men [3].

Allelic candidate gene, studies provide the most suitable method for locating genes of small effect contributing to complex genetic disorders, such as migraine [4,5]. In particular, association studies are most powerful when a plausible candidate gene and a sequence variant with potential functional relevance is examined [6].

Mutations in various ion channel genes are responsible for neuromuscular and other neurological disorders. Inherited ion channel mutations or "channelopathies" are increasingly found to be the cause of various neurological disorders in humans (see review [7]). In familial hemiplegic migraine (FHM), a rare subtype of migraine with aura, mutations in the *CACNA1A *gene (localised at C19p13) have been found (FHM1) [8]. This gene codes for the alpha1A subunit of the neuronal voltage-dependent P/Q-type calcium channel. Recently a second gene, *ATP1A2 *(FHM2) (localised at C1q23), was implicated in some FHM families [9]. The *ATP1A2 *ion channel gene, codes for the alpha2 subunit of the Na^+^, K^+ ^ion ATPase pump. These findings of mutations in these genes have focused attention on central nervous system ionic channels and helped to better understand FHM pathophysiology [10], where the best genetic evidence providing molecular insight into migraine still comes from the mutations detected in the rare form of migraine with aura; FHM [11]. The *CACNA1A *and *ATP1A2 *genes have both previously been tested in the common forms of migraine, but no new mutations or the FHM mutations were detected in these MA/MO affected samples [12-14]. Since FHM2-*ATP1A2 *is partly a potassium channel gene and is localised nearby to the potassium channel KCNN3, it may be interesting to investigate this gene in the common forms of migraine. In general, potassium (K^+^) channels set the resting membrane potential and regulate the action potential, whereby they control neuronal excitability. The small conductance (SK) calcium (Ca^2+^) activated K^+ ^channels are responsible for the "after-hyperpolarization" of neurons, which follows a train of action potentials, being activated by the increase in neuronal Ca^2+ ^[15]. The *KCNN3 *gene (C1q21.3) (a neuronal small conductance calcium-activated potassium channel, localised close to FHM2-*ATP1A2*;C1q23) encodes a protein of 731 amino acids containing two adjacent polyglutamine arrays (encoded by CAG repeats) in its N-terminal domain separated by 25 amino acids [16]. The first CAG repeat, coding for a polyglutamine stretch in exon 1 at nucleotide 88, also contains a CAA sequence anomaly after 7 repeats of CAG. However this CAG repeat region is not suitable for association studies as it is only slightly polymorphic having usually 12 CAG repeats [15,16]. The second, C-terminal, polyglutamine array, located 111 nucleotides downstream from the initial CAG repeat of the first polyglutamine stretch in exon 1, is highly polymorphic in Caucasian populations with a modal allele length of 19 and a repeat range of 10 to 28 glutamine – CAG repeats [see [15] and [16]]. Small conductance calcium-activated potassium channels (such as *KCNN3*) play a critical role in determining the firing pattern of neurons via the generation of slow after-polarization and the regulation of intracellular calcium channels [17].

*KCNN3*, localized at C1q21.3 [15], is positioned close to familial hemiplegic migraine (FHM) type 2, C1q23 [9,18], which is a severe autosomal dominant type of MA [1]. In 1999, Austin *et al *[19] suggested a mechanistic analogy for the *KCNN3 *polymorphism may be the small polyglutamine number variations in the calcium channel α1a subunit, encoded by CAG expansions in *CACNA1A *(a calcium channel implicated in FHM1) [8] which are thought to cause Spinocerebellar ataxia type 6 (SCA6) [20] by loss of channel function mechanism [21].

Polymorphic CAG repeats in the *KCNN3 *channels affect the regulation of intracellular calcium channels [17]. Since calcium channels regulate numerous processes critical to neuronal function including secretion of neurotransmitters [22], abnormal alterations in calcium channels can cause alterations in the release of neurotransmitters such as serotonin, norepinephrine, and dopamine, which all have been shown to be involved in migraine disease [23-25].

Given that FHM2 maps to C1q23 and *KCNN3 *localizes nearby at C1q21.3, it may be important to examine the prevalence of the second (highly polymorphic) *KCNN3 *CAG polymorphism in populations affected and unaffected with migraine. In the current study, we investigated the possibility of an association between migraine (MA and MO affected) and the second (more 3') CAG repeat polymorphism length variation within the *KCNN3 *gene, using a case-control study of unrelated Australian Caucasian migraine patients and ethnically matched controls.

## Methods

### Subjects

This study was approved by the Griffith University Ethics Committee and all subjects who participated gave informed consent. The subjects chosen were of Caucasian origin and were categorised based on the diagnostic criteria specified by the International Headache Society (IHS) [1]. Migraine individuals were diagnosed as having migraine with aura (MA) and migraine without aura (MO) from questionnaires and interviews conducted by an experienced clinical neurologist (JM).

In total, 423 DNA samples from unrelated individuals were analysed, of which 202 consisted of migraine patients and 221 non-migraine controls. Of the affected group, 90% of patients had a known family history of migraine, or at least one affected first degree relative. Clinically, the affected group had an average age of approximately 18 yrs disease onset, while average duration of migraine was 20 hours and frequencies of approximately 30 attacks per year. The unaffected control group was recruited from the same geographical location (East Coast of Australia) as the affected group and was matched to the case samples for variables of age (± 5 years), gender and ethnicity (Caucasian). This reduces the possibility of spurious results due to underlying population stratification. Individuals that reported being affected with known migraine comorbid conditions such as mental illness (eg. depression) and cardiovascular disease (eg. stroke) were excluded from the test groups.

### Genotyping

Genomic DNA was isolated from whole blood by a standard salting out procedure [26,27]. DNA fragments containing the second *KCNN3 *CAG polymorphism were amplified by PCR using the oligonucleotides published by Austin *et al*, 1999 [19]. The sense primer sequence was 5'-CAG CAG CCC CTG GGA CCC TCG C-3', and the anti-sense 5'-GGA GTT GGG CGA GCT GAG ACA G-3'. PCR constituents consisted of the following; 1X buffer J (Master Amp™, EPICENTRE^® ^Technologies), 0.2 μM each of forward and reverse primers, 1 unit of *Taq *polymerase and 40 ng of genomic DNA, mixed with H_2_0 in a final volume of 10 ul. Thermal cycler parameters consisted of 1 cycle at 95°C for 4 min, for initial denaturation, followed by 35 cycles of denaturation for 1 min at 94°C, primer annealing at 55°C for 1 min and primer extension at 72°C for 30 sec. Final PCR extension consisted of 72°C for 2 min [19].

The DNA samples were genotyped using an ABI PRISM™ 310 Genetic Analyzer and a FAM fluorescent dye labelled forward primer (utilized in the PCR reaction). Raw data was imported into the ABI PRISM™ Genotyper v2.0 DNA Fragment Analysis Software, whereby genotypes were individually called. Independent quality control analysis, performed by a laboratory technician, for the CAG variant consisted of PCR and genotyping of a random selection of 50 cases and controls to test for any differences between initial genotype data.

### Statistical analysis

Allele frequencies were calculated from observed genotype counts for the *KCNN3 *polymorphism. The frequencies for the *KCNN3 *gene variant were initially assessed for association with migraine using standard contingency table analysis, incorporating the chi-squared test of independence. CLUMP analysis [28], useful for association testing when markers produce sparse contingency tables, was applied to test differences in the allelic distribution between the groups of migraine and controls patients. A two-tailed type I error rate of 5% was chosen for the analyses. Data were analysed by methods described previously, based on the mode of distribution of alleles, with a modal repeat length of 19 CAG repeats [16] and the hypothesis of an association between migraine and larger repeats (>19) analysed by chi-square analysis. Allele sizes were divided into long and short groups with respect to being greater than 19 repeats or equal to and less than 19 CAG repeats in length.

## Results

**Figure 1:**
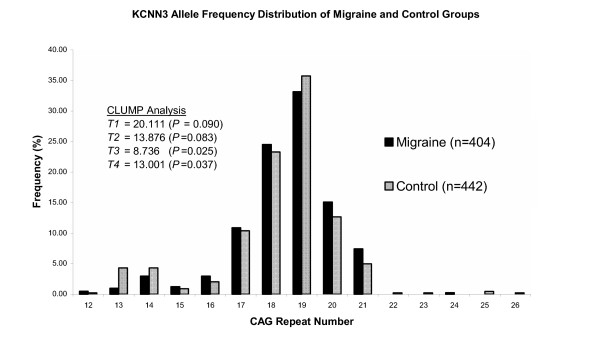
Distribution of allele frequencies by CAG repeat number for the second CAG polymorphism of the *KCNN3 *gene observed in migraine and control groups.

**Figure 2:**
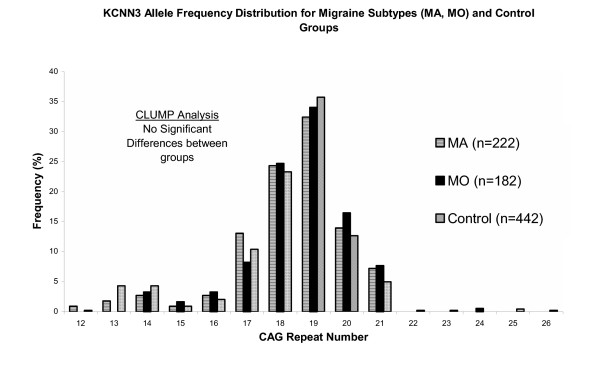
Distribution of allele frequencies by CAG repeat number for the second CAG polymorphism of the *KCNN3 *gene observed in migraine subtypes (MA and MO) and control groups.

The distribution of allele frequencies for migraine patients compared to controls are displayed in Figure 1, with the distributions of allele frequencies for MA patients, MO patients and controls displayed in Figure 2. Both figures show CAG repeat number along the X-axis versus frequency of distributions (%) displayed along the Y-axis. The repeat range is 12 to 24 repeats in migraine patients, not including the repeat lengths of 22 and 23. For the controls the range is 12 to 26 repeats, except for the 24 CAG repeat size. This displays the highly polymorphic nature of the second CAG expansion within the *KCNN3 *gene. No significant differences in allelic distributions were observed between the migraine and control groups for standard chi-square analysis (*P *= 0.090) (Figure 1). Clump analysis, for normal chi-square, also showed no significant difference reported (T1 = 20.111, *P *= 0.090). However for chi-square clumped 2×2 table analysis (obtained by clumping the columns of the original table to maximise the chi-squared value) [28], between migraine and controls, slightly significant differences were detected within the test data (T4 = 13.001, *P *= 0.037) (Figure 1). This indicates that there maybe a slight significant difference between the case-control populations with regards to clumped 2×2 table *KCNN3 *allele distributions. The T3 statistic was also positive for CLUMP analysis (*P *= 0.025) (Figure 1), although, Sham and Curtis [28] recommends that either normal chi-squared (T1) or chi-squared for "clumped" 2×2 table (T4) analysis should be used. The other two statistics (T2, T3) may lack power to detect association in data sets studied, whereas T1 and T4 perform similarly well [28].

When dividing migraine affected samples into the subtypes MA and MO, distribution of alleles did not differ significantly between MA patients and controls (*P *> 0.05) or MO patients and controls (*P *> 0.05) (Figure 2). Analysis was also performed by distributing the alleles into ≤19 (short) and >19 repeats (long) (Table I), described as per Chandy *et al*, 1998 [16]. The hypothesis of the presence of very long CAG repeats might be expected for a disorder relating to trinucleotide repeat expansion [16]; this was tested by chi-square analysis. Migraine probands showed no significant difference in allelic length distribution of the CAG repeats compared with controls (*P *= 0.15). Though the number of long CAG repeats (>19 repeats) was higher in the migraine group (92 compared to 83 for controls, Table 1) and the incidence of the CAG short repeat (<19 repeats) was higher in the control (359) group than in the migraine (312) group. Similarly, the allele frequency distributions for the migraine subgroups, MA and MO compared to controls did not approach significance (*P *= 0.46 and *P *= 0.09, respectively), or between MA vs MO (*P *= 0.40) (Table I).

## Discussion

Polyglutamine disorders are due to CAG repeat expansions that cause a toxic gain of function of mutant expanded proteins, in which protein misfolding, interference with DNA transcription and RNA processing, activation of apoptosis and dysfunction of cytoplasmic elements have all been invoked in the toxic process. [29]. These CAG expansions have been involved in a number of neurological diseases, including Huntington's disease (HD), Dentatorubralpallidoluysian atrophy (DRPLA), Kennedy's disease and Spinocerebellar ataxia (SCA) 1–3, 6–7, 12 and 17 [29].

Migraine is a polygenic multifactorial disease influenced by genetic and lifestyle characteristics. At present the mode(s) of inheritance is unclear and the type and number of migraine genes involved in the disease is not known. Since both FHM and familial typical migraine (FTM) display some clinical overlap [1], it is thought that the more prevalent FTM may also be a channelopathy [30]. The *CACNA1A *gene, involved with FHM1 [8] controls a number of fundamental neuronal processes including mediation and release of neurotransmitters such as serotonin [31]. The *KCNN3 *gene acts to regulate the firing pattern of neurons via the generation of slow after-polarization and the regulation of intracellular calcium channels [17]. Patients with migraine have autonomic nervous system dysfunction and altered levels of neurotransmitters. Therefore, decreases in activity and regulation of intracellular calcium channels, that may be caused by CAG repeat expansions in the *KCNN3 *gene, would result in decreased levels of neurotransmitters (such as serotonin) and autonomic nervous system dysfunction. These states appear to be analogous to those found during migraine headache in that decreases in plasma levels of serotonin would essentially act to constrict the arterioles, therefore causing dilation of the larger arteries, possibly causing pain [32,33].

Wittekindt *et al*, 1998 [15] reported that the *KCNN3 *gene is a good candidate for schizophrenia and bipolar disorder (BD), as well as for other neurological disorders. This includes migraine, a painful neurological disease that affects 33% of women and 13.3% of men [3].

However, even though there was a slight indication of significance in one analysis (clump T4 statistic) [28], overall the results of this study proved that there was no statistically significant association between allelic frequencies of migraine and non-migraine patients. Also no significant difference in allelic frequencies was observed in the migraine subtypes, MA and MO when compared to the control population. There have been many contradicting studies that have shown a significant over-representation or non-significant representation of long CAG repeats in the *KCNN3 *gene in patients with schizophrenia and bipolar disease compared to population controls [see [34]]. In a meta-analysis of association studies for schizophrenia and bipolar disorder with the CAG-repeat length in KCNN3, the results demonstrated that the risks for both of these disorders were largely, if not entirely, independent of the CAG-repeat in the KCNN3 gene [34]. This study examined CAG repeat lengths (the second, C-terminal, polyglutamine array in exon 1 of KCNN3) in migraine patients and also found no significant evidence to suggest long CAG repeats (>19) are over-represented in patients with migraine compared to controls, even though there was a higher incidence reported for long CAG repeat (>19 repeats) in the migraine group compared to the controls (Table I), it did not reach statistical significance (*P *< 0.05). However, a recent study by Mossner *et al*, 2005 [35], reported an association between the second highly polymorphic polyglutamine stretch and migraine. In this study the authors found allele 15 to be associated with migraine (*P *= 0.027; total migraine) [35]. Allele 15 is extremely rare with a 1.6% frequency in MA, 1.5% in MO and a 0.2% control frequency found in Mossner et al's study [35]. In our investigation of the second KCNN3 – CAG repeat we found a frequency in allele 15 of 0.9% in MA (only 2 out of 222 alleles), 1.65% in MO (3 out of 182 alleles) and 0.9% in controls (4 out of 442 alleles) (see distribution of alleles, Figures 1 and 2). From the two studies it can be seen that this allele is too rare to make any assumptions of whether it is migraine related. A population base far greater in number is needed in order to obtain meaningful data from allele 15. Also, the fact that this study utilised samples that were all carefully diagnosed following IHS guidelines [1], including all samples from the migraine unaffected-control population and Mossner et al, 2005 [35] did not, leaves their positive results questionable. Mossner et al had made a migraine-unaffected assumption with more than half their controls (119/232 samples). These individuals that were utilised as controls, were an undiagnosed, anonymous blood donor group [35], that were not directly interviewed or diagnosed according to the International Headache Society guidelines [1]. This could have a tremendous impact on the outcomes of their study, due to the rarity of allele 15 in the second CAG repeat of exon1 in *KCNN3*.

The present association study was conducted in carefully diagnosed (according to IHS guidelines [1]), age, sex and ethnicity matched case-control populations. However the results provided no evidence that *KCNN3 *gene confers susceptibility to the subtypes of migraine (MA and MO), or common migraine.

## Conclusion

We conclude that our data does not confer with Mossner *et al*, 2005 [35], providing no evidence that a variation at the polymorphic, second CAG repeat locus, in the *KCNN3 *gene, influences susceptibility to migraine, or to the migraine subtypes, MA and MO.

## List of abbreviations

*KCNN3 *neuronal small conductance calcium-activated potassium channel, subfamily N, member 3, MA migraine with aura, MO migraine without aura.

## Competing interests

The author(s) declare that they have no competing interests.

## Authors' contributions

RC carried out the optimisation of this molecular genetic study and drafted the manuscript. JS carried out PCR and genotyping. RL carried out statistical analysis. MO carried out DNA sample preparation for the populations. JM carried out patient clinical diagnosis. LG conceived of the study, and participated in its design and coordination and helped to draft the manuscript. All authors read and approved the final manuscript.

## Pre-publication history

The pre-publication history for this paper can be accessed here:



## Figures and Tables

**Table 1:** Long (>19 repeats) and short (≤19 repeats) alleles: CAG repeat number in *KCNN3*; analysis performed utilising the comparison of long and short allele repeat numbers between migraine and control populations.

**Allele Size**		
Group	Short (≤19rpts)	Long (>19rpts)

MA	175	47
MO	137	45
Migraine (Total)	312	92
Control	359	83

* Analysis of long and short alleles: no significant differences were obtained between migraine vs control groups (*P *= 0.15), MA vs controls (*P *= 0.46), MO vs controls (*P *= 0.09), or between MA vs MO (*P *= 0.40)
